# Probability expression for changeable and changeless uncertainties: an implicit test

**DOI:** 10.3389/fpsyg.2014.01313

**Published:** 2014-11-13

**Authors:** Yun Wang, Xue-Lei Du, Li-Lin Rao, Shu Li

**Affiliations:** ^1^Key Laboratory of Behavioral Science, Institute of Psychology, Chinese Academy of SciencesBeijing, China; ^2^University of Chinese Academy of SciencesBeijing, China

**Keywords:** verbal probability, numerical probability, changeable uncertainty, unchangeable uncertainty, changeability feature

## Abstract

“Everything changes and nothing remains still.”We designed three implicit studies to understand how people react or adapt to a rapidly changing world by testing whether verbal probability is better in expressing changeable uncertainty while numerical probability is better in expressing unchangeable uncertainty. We found that the “verbal-changeable” combination in implicit tasks was more compatible than the “numerical-changeable” combination. Furthermore, the “numerical-changeless” combination was more compatible than the “verbal-changeless” combination. Thus, a novel feature called “changeability” was proposed to describe the changeable nature of verbal probability. However, numerical probability is a better carrier of changeless uncertainty than verbal probability. These results extend the domain of probability predictions and enrich our general understanding of communication with verbal and numerical probabilities. Given that the world around us is constantly changing, this “changeability” feature may play a major role in preparing for uncertainty.

## Introduction

Uncertainty exists in many aspects of our daily lives; thus, we use probability predictions to mitigate and prepare for uncertain events. For example, we may wonder if air quality will be acceptable tomorrow, if the stock market will continue to rise, if a hurricane will occur in a specific place, etc. We need to be able to predict the probability of uncertain events to guide our decisions.

Because perceptions of risk and uncertainty are affected by individual's worldview and experience (Fox and Irwin, 1998), predicting the likelihood of an event depends on how a person views uncertainty. There are two views on uncertainty. One view indicates that uncertainty is changeable, whereas the other view states that uncertainty is predetermined and changeless. Both views can be traced back to religious perspectives on destiny and life, including Buddhism and Islam. Buddhism views life as a dynamic and ever-changing process, in which beings experience a succession of lifetimes as one of many possible life forms (Keown, 2000). In this dynamic process, individuals' fate changes according to their karma (the actions or deeds conducted by an individual in their life). Good karma leads to good fortune, while bad karma results in bad fortune. “A butcher becomes a Buddha the moment he drops his cleaver” is an example of Buddhism's emphasis on the changeable nature of uncertainty. In contrast, some groups believe in the doctrine of predetermination. For example, the Al-Jabriyyah sect of Islam believes that everything in the world is preordained and nothing can happen unless permitted by Allah, i.e., the fate of an individual has been predetermined and cannot be changed by free will. Despite this belief, people will not have knowledge about future events until such events occur. Thus, even if our lives have been predetermined and are changeless, the future is still uncertain.

In addition to religious perspectives, two views on uncertainty can be found in two prediction theories: *I Ching* (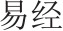) and modern probability theory. The *I Ching*, also known as the *Book of Changes*, emphasizes the importance of predicting the future from a changing perspective. The book discusses changes between *yin* and *yang*; where *yin* becomes *yang* when *yin* reaches the extreme and vice versa (Wei, 1939). According to this theory, everything in the universe is changing. Thus, people should consider this variability and not regard the outcomes of future events as unalterable when predicting the likelihood of events. For example, the Jiji (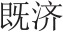) Hexagram in *I Ching* states that “a good beginning may bring a bad ending” (Wang and Ren, 1993). This statement denotes that an enterprise can begin with good fortune but end in disaster, thus, situations change over time. Therefore, the uncertainty implied by *I Ching* changes according to circumstances. In contrast, modern probability theory, which is rooted in the attempts of Gerolamo Cardano to analyze games of chance in the sixteenth century, applies mathematical thinking to prediction theory and assigns unchangeable predictions to uncertain outcomes. Modern probability theory considers uncertain events as random, exhibiting a sequence of certain patterns (e.g., the law of large numbers) that can be predicted if repeated many times. Thus, the potential outcomes of random events are limited to mathematical laws and the likelihoods of such outcomes are stationary and fixed. For example, tossing a coin will result in one of two equally possible outcomes: heads or tails. The probability of a heads outcome is 1/2, which will not change.

Languages are carriers and instruments of thought. Theoretical prediction outcomes are ultimately expressed by language. Different views on uncertainty that are embodied in different religions and theories are also reflected in language. People often use two types of probabilistic statements to transmit uncertainty: verbal probability (e.g., possible) and numerical probability (e.g., 40%). When people perceive uncertainty as changeable, the selected probability expression (verbal or numerical) allows for potential changes. In this situation, verbal probability is more capable of expressing the desired changeability than numerical probability. Previous studies have shown that different verbal probabilities can be represented by membership functions over the numerical [0, 1] probability interval, which has a location and shape that varies with individuals and contexts (Wallsten et al., 1986; Weber and Hilton, 1990). For example, a membership function may represent “possibility” over a numerical probability range of 30–60%, with 40% as the best point of equivalence that contains the maximum value (generally one) of the function. However, in other contexts, both the numerical probability range and the best point of equivalence can differ. The meanings of verbal probabilities change according to circumstances, thus allowing individuals flexibility in expressing changeable uncertainty. In contrast, when people perceive uncertainty as predetermined and changeless, the selected probability expression has a stable and explicit interpretation with insignificant variation. In this case, numerical probability, which does not satisfy the property of a changeable membership function, is an ideal expression because the approach is objective and precise. A previous study (Windschitl and Wells, 1996) demonstrated that numeric measures of uncertainty tend to move people toward deliberate and rule-based thinking. This tendency can increase numerical probability use when people believe that certain rules predetermine uncertainty. The above reasoning process leads to the following assumptions: verbal probability expression reflects the “changeable” view of uncertainty, whereas numerical probability expression reflects the “predetermined and changeless” view of uncertainty.

A living being is seen as a form of process in the universe. A living being can maintain its own identity and endure in time while continuously undergoing change (McCracken, 1952). The animate is always in motion and exhibits physical and psychological changes, unlike the inanimate. According to this view, the recent finding that verbal and numerical probabilities are the preferred manner of expressing animate and inanimate uncertainties, respectively (Du et al., 2013), can be seen as empirical evidence to defend our assumptions. Nevertheless, although the animate/inanimate may serve as proxy measures for changeable/changeless events, animate/inanimate is not the only representation of changeable/changeless. The bidirectional association between verbal/numerical probabilities and animate/inanimate uncertainties found in Du et al. (2013) experiments is not about changeability in general, i.e., animateness is not equated with changeability. Additional studies are needed to test our assumptions about the association between verbal/numerical probabilities and changeable/changeless uncertainties outside the realm of living vs. nonliving agents.

The bidirectional association reported by Du et al. (2013) was implemented and observed with explicit assessment procedures. Implicit assessment uses involuntary behavior, which is more resistant to deliberative control or faking than self-reported measures (Kim, 2003; Fiedler and Bluemke, 2005), to measure attitudes about which respondents might be unaware (Greenwald and Banaji, 1995; Vartanian et al., 2004). Compared to explicit self-report, Greenwald et al. (1998) believed that implicit assessment, which measures beliefs through indirect or implicit means, avoids participants' self-disguise and measures in-depth attitudes of which people are not aware (Greenwald et al., 1998). Because the association between probability expression and uncertainty type is not obvious at the superficial level, we investigated this association with implicit assessment. Thus, the present study further examined associations between probability expression and uncertainty type by using an implicit assessment procedure.

## Overview

Three studies and a pilot study examined if verbal and numerical probabilities were closely associated with changeable and changeless uncertainties. In the pilot study, we found that people perceive animate objects as changeable and inanimate objects as changeless. Then, we used an implicit paradigm (i.e., a font preference task) to test the bidirectional relationship between verbal/numerical probabilities and changeable/changeless uncertainties in scenarios where the same uncertain event occurred to animate/inanimate objects (Study 1). We found that verbal probability was likely to be used to predict the likelihood of events that affect animate objects but fails to find an association between inanimate objects and numerical probability. Because Arabic numerals (e.g., 50%) may not be as favorable as real word characters (e.g., fifty percent) for the font-type rating task, we modified the experimental material in Study 1 by replacing the Arabic numerals with Chinese characters (Study 2). We found that people prefer verbal and numerical probability expressions when the uncertainties are from animate beings and inanimate objects. We further investigated associations between verbal/numerical probabilities and changeable/changeless uncertainties in scenarios in which the same uncertain event occurred to the same inanimate object but with different changeable situations using the same implicit task (Study 3). We found that people prefer verbal probability expressions when uncertainties are changeable and that people prefer numerical probability expressions when uncertainties are changeless. The present study was approved by the Institutional Review Board of the Institute of Psychology, Chinese Academy of Sciences.

## Pilot study

Because living things continuously undergo changes (McCracken, 1952), we used animate objects/living things to represent the perception of changeable uncertainty and inanimate objects to represent the perception of changeless (or at least less changeable) uncertainty to test the bidirectional association between verbal/numerical probabilities and changeable/changeless uncertainties. This manipulation is supported by research on the distinction between animate and inanimate objects. People who focus on the psychological properties of animate objects, which are usually indeterminate, consider their reactions unpredictable, whereas people who focus on the physical properties of inanimate objects, which are deterministic, tend to believe that their reactions are predictable (Gelman and Spelke, 1981). Furthermore, the pilot study confirmed that participants consider animate objects changeable [93.6% (29/31)] and inanimate objects changeless [90.32% (28/31)]. In Study 1, we tested whether the likelihood of an event happening to animate or inanimate objects would be closely related to verbal and numerical probabilities.

## Study 1

In this study, participants evaluated the beauty of several font types in a sentence that described the likelihood of an event happening to an animate or inanimate object through verbal or numerical probability expression. Close associations may facilitate information processing (as in an Implicit Association Test), and conceptual processing fluency leads to positive evaluations (Reber et al., 1998; Lee and Labroo, 2004). Lee and Labroo (2004) extended the processing fluency model to examine the effect of conceptual fluency on attitudes, and found that increased perceptual fluency leads more favorable attitudes toward a brand among consumers (Lee and Labroo, 2004). Reber et al. (1998) examined the effects of perceptual fluency on affective judgments and found that participants judged targets as prettier if they were preceded by a matching, rather than nonmatching, prime. Perceptual fluency increases judgments of prettiness and liking (Reber et al., 1998). Based on this knowledge, we believed that participants in the font-type rating task would process the sentences of compatible objects more fluently and evaluate them as more beautiful compared to the sentences of incompatible objects. Thus, we hypothesized that if people implicitly believed that changeable uncertainty was compatible with verbal probability and that changeless uncertainty was compatible with numerical probability, the participant would judge the most beautiful font type in the sentence by verbal probability in combination with information on an animate being (or numerical probability with an inanimate object). We performed the following experiment to test this hypothesis.

### Method

A total of 105 participants completed Study 1 and were compensated with a gift (worth CNY 2.2). The implicit measure was a font-type rating task (Table 1). Four conditions, person–verbal (PV), person–numerical (PN), computer–verbal (CV), and computer–numerical (CN), tested the compatibility between verbal/numerical probabilities and the uncertainties of animate/inanimate objects (changeable/changeless uncertainties). In each condition, a sentence that described the likelihood of an event occurring to a person or a computer in verbal or numerical probability was printed in 4 different fonts. Participants were asked to indicate the beauty of each font type on a scale of 74 mm, which was anchored at extremes with the labels “not beautiful at all” and “extremely beautiful.” The rating was directly transformed into numerical values between 0 and 74. Each participant evaluated all 4 conditions in an order that was counterbalanced across participants. This study was a 2 × 2 within-subjects design, with probability expression (verbal, numerical) and uncertainty type (changeable, changeless) as within-subjects factors.

**Table 1:**
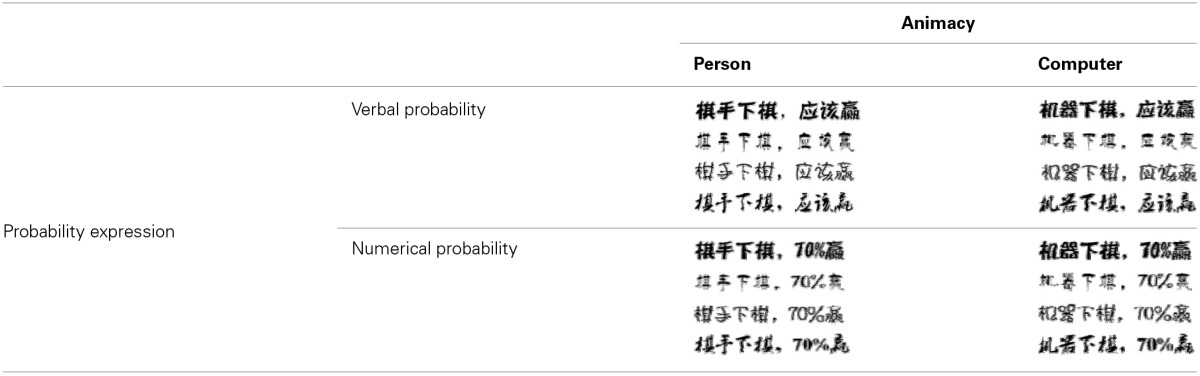
**The four font-type rating task conditions: person-verbal (PV), person-numerical (PN), computer-verbal (CV), and computer-numerical (CN)**.

*The English meanings of the four cells are as follows: “the chess player has a good chance to win the game” (PV); “the computer has a good chance to win the game” (CV); “the chess player has a 70% chance of winning the game” (PN); “the computer has a 70% chance of winning the game” (CN)*.

### Results and discussion

**Figure 1:**
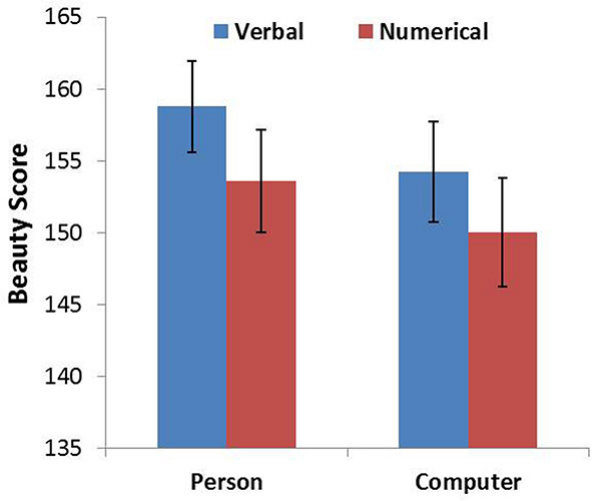
**Beauty score as a function of probability expression for person and computer**. Error bars denote standard errors.

For each animacy–probability pairing condition, the four ratings corresponding to the four different font types were combined into a single variable that represented overall beauty. An ANOVA was conducted on the newly generated variable with probability expression and animacy as within-subjects factors. The analysis revealed main effects for probability expression [*F*_(1, 104)_ = 5.640, *p* = 0.019, η^2^ = 0.051] and animacy [*F*_(1, 104)_ = 4.486, *p* = 0.037, η^2^ = 0.041]. *Post-hoc* analysis showed that participants rated the font types in the verbal probability sentences as more beautiful than the numerical probability sentences and that participants rated the font types in the chess player sentences as more beautiful than the computer sentences (Figure 1). However, we did not find an interaction between probability expression and animacy. Our results showed that the font type beauty score for verbal probability expression was higher than numerical probability expression for both the chess player and computer sentences. Thus, we did not find that the beauty score in the CN (computer–numerical) condition was higher than that in the CV (computer–verbal) condition.

The results provide evidence for our prediction that the animate being (a person) and verbal probability combination would be more compatible than the animate being and numerical probability combination. This result implies that participants implicitly associate the uncertainty of the animate being (changeable uncertainty) with the verbal probability expression. We did not find that the association between inanimate objects (changeless uncertainty) and numerical probability is more compatible than the association between inanimate objects and verbal probability. A *post-hoc* explanation for this result is that the font-type rating task may have not worked as well with Arabic numerals.

In summary, Study 1 provides implicit evidence for our prediction that verbal probability is more likely to be used when predicting, describing, and measuring changeable uncertainty.

## Study 2

Study 1 demonstrated an implicit association between verbal probabilities and animate beings. However, Study 1 failed to find an association between an inanimate object and numerical probability. Study 2 was conducted to examine if the failure to detect Study 1's computer–numerical compatibility in the font-type rating task was caused by the use of Arabic numerals. As such, in Study 2, we modified the experimental material and replaced Arabic numerals (70%) with Chinese characters (seventy percent in Chinese).

### Method

A total of 103 participants completed Study 2 and were compensated with a gift (worth CNY 2.0). The font-type rating task was also the implicit measure in Study 2. Similar to Study 1, we designed four conditions (Table 2). In each condition, a sentence that described the likelihood of an event happening to a person or computer in verbal or numerical probability was printed in 4 fonts. In contrast to Study 1, all sentences were written in Chinese characters. Participants were asked to rate the beauty of each font type on a scale of 74 mm. The four conditions in this study were counterbalanced across participants. This study was a 2 (probability expression) × 2 (uncertainty type) within-subjects design.

**Table 2:**
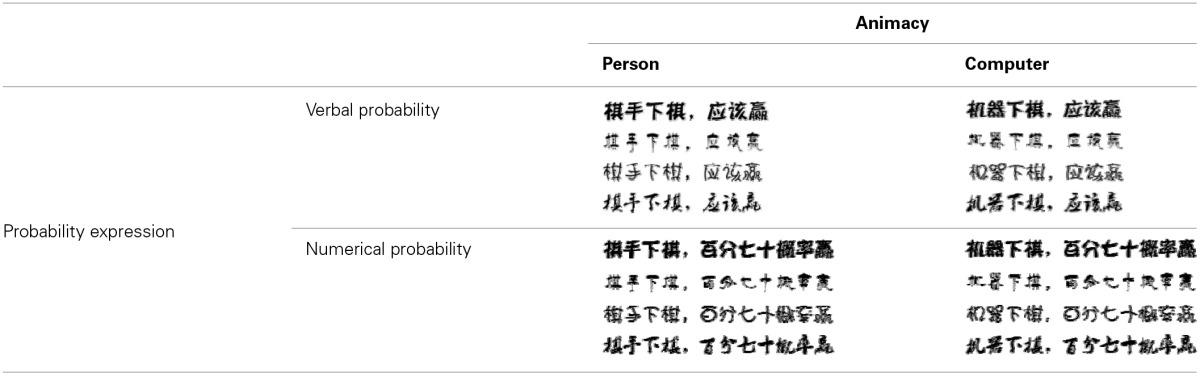
**The four font-type rating task conditions: person-verbal (PV), person-numerical (PN), computer-verbal (CV), and computer-numerical (CN)**.

*The English meanings of the four cells are as follows: “the chess player has a good chance to win the game” (PV); “the computer has a good chance to win the game” (CV); “the chess player has a seventy percent chance of winning the game” (PN); “the computer has a seventy percent chance of winning the game” (CN)*.

### Results and discussion

**Figure 2:**
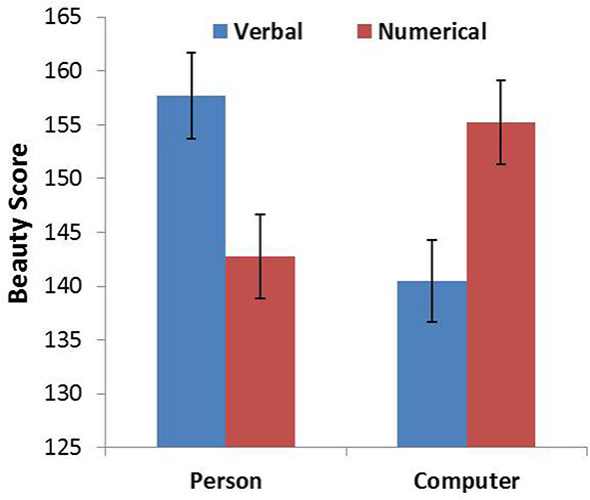
**Beauty score as a function of probability expression for person and computer**. Error bars denote standard errors.

As in Study 1, we summed the rating score corresponding to the four different font types for each animacy-probability pairing condition, which represented the overall beauty of the font types for each condition. An ANOVA was conducted with probability expression and animacy as the within-subjects factors. The analysis revealed that the main effects of probability expression and animacy were not significant. However, there was a significant interaction between probability expression and animacy [*F*_(1, 102)_ = 61.533, *p* < 0.001, η^2^ = 0.376]. Simple effects analyses indicated that the beauty score in the PV (person–verbal) condition was significantly higher than the beauty score in the PN (person–numerical) condition (*p* < 0.001), whereas the beauty score in the CN (computer–numerical) condition was significantly higher than the beauty score in the CV (computer–verbal) condition (*p* < 0.001; Figure 2).

The results of Study 2 provided evidence for our hypothesis that the combination between an animate being and verbal probability would be more compatible than the combination between an animate being and numerical probability. We also found that the combination between an inanimate object and numerical probability was more compatible than the combination between an inanimate object and verbal probability in the scenario that did not use Arabic numerals. This finding implies that participants closely associate the uncertainty of animate objects (changeable uncertainty) with verbal probability expression and that there is a close relationship between the uncertainty of inanimate objects (changeless uncertainty) and numerical probability.

In sum, Study 2 provides evidence for our hypothesis about the verbal probability and animate objects (changeable uncertainty) association and the numerical probability and inanimate objects (changeless uncertainty) association without using Arabic numerals. It is worth noting that we used animate/inanimate objects as proxies of changeability in this study.

## Study 3

As animate/inanimate is not the only representation of changeable/changeless, we directly manipulated changeability instead of using animate/inanimate objects as proxies of changeability to more broadly test the associations in Study 3. We designed two “changeability” scenarios: the water (river/pool) scenario and the die (rolling/settled) scenario. In this study, we asked participants to evaluate the beauty of various font types in sentences that described two non-Arabic inanimate scenarios. Table 3 shows the sentences that described the likelihood that the water level of a river or pool would rise through verbal or numerical probability expression. Table 4 shows the sentences that described the likelihood of obtaining an odd number from a rolling or settled die through verbal or numerical probability expression. Rivers flow, whereas pools are still. Furthermore, a rolling die is full of change, whereas settled dice are stationary. Thus, we postulated that participants would judge the most beautiful sentence font types by verbal probability in combination with the river/rolling die (or numerical probability with a pool/settled die). We performed the following experiment to test this hypothesis.

**Table 3:**
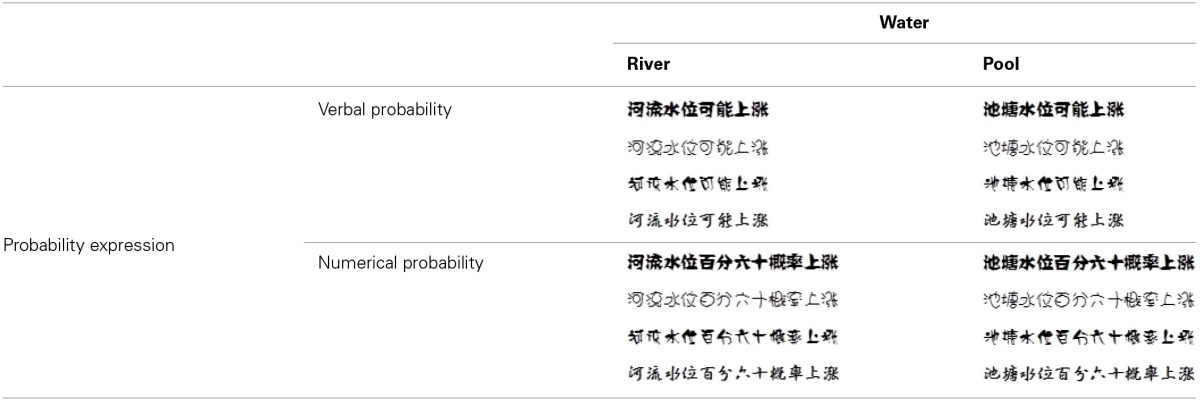
**The four font-type rating task conditions: river-verbal (RV), river-numerical (RN), pool-verbal (PV), and pool-numerical (PN)**.

*The English meanings for the four cells are as follows: “the water level of a river may rise” (RV); “the water level of a pool may rise” (PV); “the water level of a river has a sixty percent chance of rising” (RN); “the water level of a pool has a sixty percent chance of rising” (PN)*.

**Table 4:**
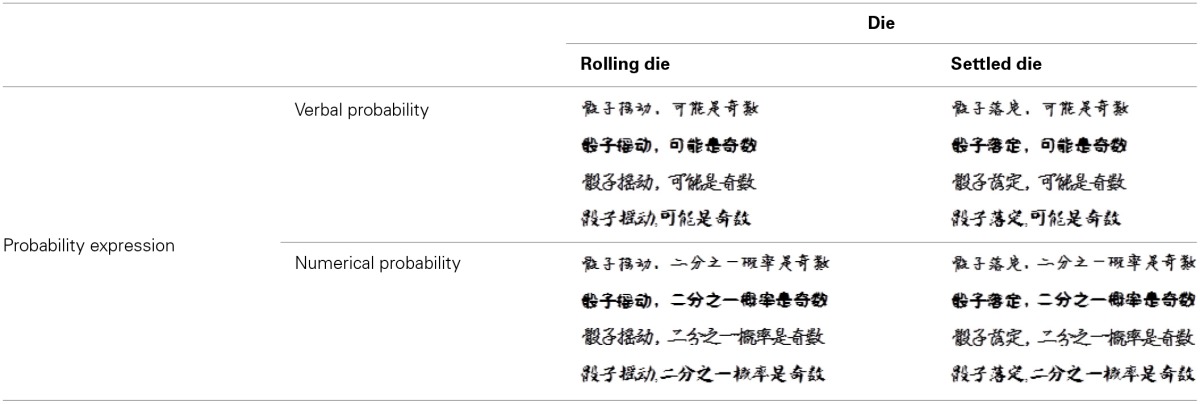
**The four font-type rating task conditions: rolling die-verbal (RV), rolling die-numerical (RN), settled die-verbal (SV), and settled die-numerical (SN)**.

*The English meanings of the four cells are as follows: “an odd number can be obtained for a rolling die” (RV); “an odd number can be obtained for a settled die” (SV); “an odd number has a one-half chance of appearing for a rolling die” (RN); “an odd number has a one-half chance of appearing for a settled die” (SN)*.

### Method

A total of 97 participants completed Study 3 and were compensated with a gift (worth CNY 3.8). The implicit measure for Study 3 was the font-type rating task. For the water scenario, we designed four conditions: river–verbal (RV), river–numerical (RN), pool–verbal (PV), and pool–numerical (PN; Table 3). Additional 4 conditions were designed for the die scenario to test the compatibility between verbal/numerical probabilities and changeable/changeless uncertainties: rolling die–verbal (RV), rolling die–numerical (RN), settled die–verbal (SV), and settled die–numerical (SN; Table 4). For each condition, there was a sentence that described the likelihood of an event happening to the 4 objects (river/pool and rolling/settled die) in verbal or numerical probability, which was printed in 4 fonts. Similar to the two former studies, we asked participants to rate the beauty of each font type on a scale of 74 mm. The four conditions for each scenario were counterbalanced across participants. This study was a 2 (probability expression) × 2 (uncertainty type) within-subjects design. We also asked participants to evaluate the degree of change of the river/pool and rolling/settled die on a 7-point Likert scale (1 = “very little,” 7 = “very large”) after the font-type rating task.

### Results and discussion

Paired-sample *t*-tests showed that participants judged the river as significantly more changeable than the pool [*M*_river_ = 4.90; *M*_pool_ = 3.08; *t*_(95)_ = 9.697, *p* < 0.001] and that the rolling die was significantly more changeable than the settled die [*M*_rolling die_ = 5.46; *M*_settled die_ = 3.32; *t*_(96)_ = 8.365, *p* < 0.001]. This result was in line with our hypothesis.

**Figure 3:**
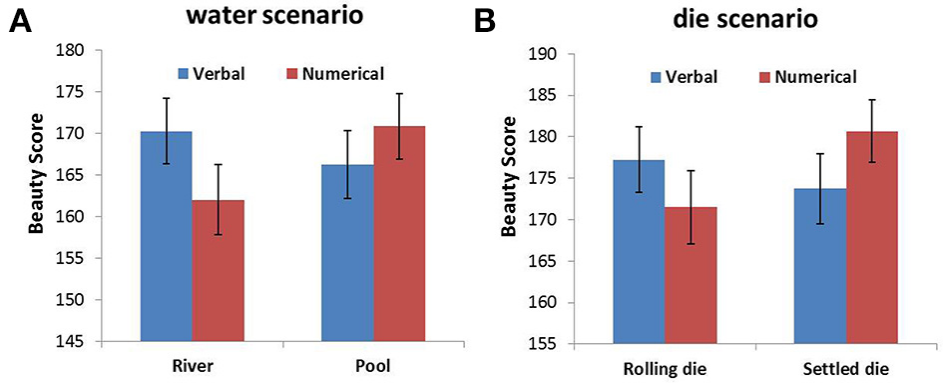
**Beauty score as a function of probability expression for objects with different changeability in the water scenario (A) and the die scenario (B)**. Error bars denote standard errors.

For the font-type rating task, we summed the rating score that corresponded to the four different font types for each changeability-probability pairing condition, which represented the overall beauty of the font type for each condition. Thereafter, an ANOVA was conducted for each scenario with probability expression and changeability as within-subjects factors. The analysis revealed that the probability expression and changeability in both scenarios had no main effects. There were significant interactions between probability expression and changeability for the water scenario [*F*_(1, 96)_ = 11.264, *p* = 0.001, η^2^ = 0.105] and the die scenario [*F*_(1, 96)_ = 7.681, *p* = 0.007, η^2^ = 0.074] (Figure 3). In the water scenario, a simple effects analysis indicated that the beauty score in the RV (river–verbal) condition was significantly higher than the beauty score in the RN (river–numerical) condition (*p* = 0.002), while the beauty score in the PN (pool–numerical) condition was significantly higher than the beauty score in the PV (pool–verbal) condition (*p* = 0.046; Figure 3A). Similar results were found for the die scenario, i.e., the beauty score in the RV (rolling die–verbal) condition was significantly higher than the beauty score in the RN (rolling die–numerical) condition (*p* = 0.038), while the beauty score in the SN (settled die–numerical) condition was significantly higher than the beauty score in the SV (settled die–verbal) condition (*p* = 0.017; Figure 3B). The sentences in the die scenario received higher beauty scores than those in the water scenario. This may be because different font types were used in the two scenarios, as shown in Tables 3, 4.

The results from Study 3 provided evidence for our hypothesis that the combination between a changeable object (a river/rolling die) and verbal probability would be more compatible than the combination between a changeable object and numerical probability. We also found that the combination between a changeless object (a pool/settled die) and numerical probability was more compatible than the combination between a changeless object and verbal probability in scenarios that did not use Arabic numerals. This finding implies that participants associate changeable uncertainty with verbal probability expressions and that the association between changeless uncertainty and numerical probability is robust.

Study 3 provides evidence for our prediction on the verbal probability and changeable uncertainty association and the numerical probability and changeless uncertainty association in the two scenarios that do not utilize living agents.

## General discussion

We investigated the associations between verbal/numerical probabilities and changeable/changeless uncertainties by using implicit assessments. The results of the three studies demonstrate that people closely associate verbal probabilities with changeable uncertainty and that people closely associate numerical probabilities with changeless uncertainty. Our findings suggest that changeability may serve as an additional semantic feature of verbal probability.

People use verbal probability more often than numerical probability in daily life (Budescu et al., 1988). Since the 1960s, the features of verbal probability have been intensely studied to examine its role and efficacy in communicating uncertainty and decision-making. Previous studies have identified three semantic features of verbal probability, which convey additional semantic information. One feature is *directionality*. Most verbal probabilities are unidirectional and fall into one of two types: positive (suggesting the occurrence of a described outcome; e.g., possible) and negative (drawing attention to its nonoccurrence; e.g., impossible; Teigen and Brun, 1999, 2000; Juanchich et al., 2010, 2013). From the directional perspective, numerical probabilities are more equivocal than verbal phrases because the directional trend of a numerical probability is undefined. The second feature is *internal/external attribution*. The internal/external attribution of uncertainty can be distinguished when verbal probabilities are placed in different syntactical structures: “I am uncertain…” reflects uncertainty that is attributed to one's state of knowledge, whereas “It is uncertain…” reflects uncertainty that is attributable to the external world (Kahneman and Tversky, 1982). Fox and Irwin (1998) suggested that the use of internal expressions of uncertainty triggers more certitude and responsibility than the use of external expressions. The final feature is *self-serving interpretation*. When using verbal probabilities to describe the likelihood of events in one's own future or the future of others, people interpret verbal chance in a self-serving manner (Smits and Hoorens, 2005). Thus, people will believe that positive events are more likely than negative events to occur in their own futures compared to the future of others, despite probability descriptions being the same. As the Zhen (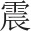) Hexagram in *I Ching* states, “thunders do not hurt him, but his neighbor had error” (Wang and Ren, 1993). These three features demonstrate that verbal probability is not just another way of expressing a quantitative probability, but is also a carrier that conveys important semantic information. The current research contributes to this literature by detecting an additional semantic feature for verbal probability, namely, “changeability,” which describes the changeable nature of verbal probability. According to our results, verbal probability is useful for describing largely changeable uncertainty, whereas numerical probability, which has an “unchangeable” feature, is suitable for describing relatively changeless uncertainty. To the best of our knowledge, this changeability feature of verbal probability is reported in this paper for the first time in the field of uncertainty communication.

The compatibility between verbal probability and animate beings/changeable objects has been confirmed even with implicit measures. Previous studies have investigated the association between animate/inanimate uncertainties and verbal/numerical probabilities using explicit procedures (Du et al., 2013). In the present study, we examined the relationship between uncertainty type and probability expression using implicit procedures. We did not use animate and inanimate objects as proxies for changeability, but instead directly manipulated changeability. Previous studies that have examined the animate–inanimate distinction have found that people often focus on the psychological properties of animate objects, which are indeterminate and varying, and on the physical properties of inanimate things, which are fixed (Gelman and Spelke, 1981). From this perspective, the distinction between animate and inanimate on changeability is apparent. Our pilot study also shows this difference. To an extent, animate and inanimate objects may be used as proxies for changeable and changeless events and the verbal probability of animate objects can further be generalized as a welcome and proper mode for communicating uncertainty in situations that are full of potential changes or variations. Furthermore, our study found a robust association between verbal/numerical probabilities and changeable/changeless uncertainties in the field of inanimate objects. Thus, changeability can describe and contrast a variety of objects outside the realm of living vs. nonliving agents. This finding suggests that the verbal probability–changeable uncertainty combination is automatic, well ingrained, and can significantly increase the positive evaluation of an irrelevant stimulus, such as font type. This study was a preliminary exploration of the associations between probability expression and uncertainty type. There may be additional factors that affect these findings in real-life settings. Future research is needed to test and verify the mechanisms between probability expression and uncertainty type.

It is important to consider several additional points. First, the primary purpose of this research was to examine if verbal probability is suited to describing changeable uncertainty and if numerical probability is suited to describing changeless uncertainty. This study directly manipulated animacy (animate/inanimate) and the changeable degree of inanimate objects. Future research may be able to manipulate other variables to further confirm our findings. Second, we did not examine the effect of appropriate communication modes on decision-making quality. Our study indicates that compatible (e.g., verbal-changeable uncertainty) combinations have a positive influence on the evaluation of font types. This effect may be caused by conceptual processing fluency (Lee and Labroo, 2004), which may affect the desirability of alternatives with uncertain outcomes in decision making. This topic is an important avenue for future research.

We live in a world of rapid change. According to the Greek philosopher Heraclitus, “everything changes and nothing remains still.” In this study, we found that verbal probability possesses a semantic feature, which we named *changeability*. This suggests that verbal probability is responsible for predicting changeable uncertainty. We were also able to identify a good match between the changeability feature and the changing nature of the real world. Verbal probability plays an important role in describing the changing world, regardless of whether the changeability feature is supported. This result may be used to explain the popular use of verbal probability in daily life (Wallsten et al., 1986; Budescu et al., 1988; Reagan et al., 1989) and the limited role of numerical probability in conveying scientific information to the public (with the exception of gene science and weather forecasting fields, wherein numerical probability is common). Therefore, although changeability is a late addition to the group of verbal probability features, it is not a simple accumulation of terminology. Changeability is important for enabling researchers and practitioners to understand how people react or adapt to a changing world. Research should continue to examine this topic to clarify its effects on decision-making under risk and uncertainty conditions. Also, people are known to make large errors when adapting their probability estimates to changes in the situation (e.g., Shaklee and Fischhoff, 1990; Yechiam and Budescu, 2006). Future studies could examine if these errors may be alleviated by the use of verbal descriptions of likelihood.

## Author's note

This research was partially supported by the National Basic Research Program of China (973 Program, No. 2011CB711000), the Knowledge Innovation Program of the Chinese Academy of Sciences (No. KSCX2-EW-J-8), the National Natural Science Foundation of China (No. 31170976; 31300843), Foundation for the Supervisor of the Beijing Excellent Doctoral Dissertation (No. 20138012501), and the Scientific Foundation of Institute of Psychology, Chinese Academy of Sciences (No. Y2CQ043005).

### Conflict of interest statement

The authors declare that the research was conducted in the absence of any commercial or financial relationships that could be construed as a potential conflict of interest.
